# Association between diagnosis of depression and health service coverage: a multilevel analysis of the National Health Survey, Brazil, 2019

**DOI:** 10.1590/S2237-96222026v35e20250666.en

**Published:** 2026-02-27

**Authors:** Flávia Oliveira da Silva, Andressa Lourenço Carvalho, Guilherme Guimarães Maia Schnepper, Léo Domingues Marchesi, Isabella Ribeiro Leite, Laura Vazarin Endo, Camila Del Valhe Sanchez Lima, Kaio Henrique Correa Massa

**Affiliations:** 1 Universidade Anhembi Morumbi, São Paulo, SP, Brasil

**Keywords:** Depression, Access to Health Services, Social Determinants of Health, Mental Health, Epidemiology, Depresión, Acceso a los Servicios de Salud, Determinantes Sociales de la Salud, Salud Mental, Epidemiología

## Abstract

**Objective:**

To analyze association between prevalence of diagnosis of depression in adults and health service coverage in Brazil’s Federative Units.

**Methods:**

This was a cross-sectional study using data from the 2019 National Health Survey. Adjusted odds ratios (OR) and 95% confidence intervals (95%CI) were calculated from multilevel logistic regression models to analyze association between diagnosis of depression and health service coverage.

**Results:**

88,531 adults residing in the 27 Brazilian Federative Units were analyzed in 2019. Higher odds of depression diagnosis were found among adults residing in Federative Units with greater Family Health Strategy coverage (OR 1.35; 95%CI 1.29; 1.42), with a greater number of family health support centers (OR 1.17; 95%CI 1.13; 1.21) and psychosocial care centers (OR 1.31; 95%CI 1.23; 1.41).

**Conclusion:**

The results of this study indicated that greater health service coverage was associated with higher prevalence of diagnosis of depression. These findings emphasized the importance of expanding and strengthening the psychosocial care network to improve diagnosis and care of individuals with mental disorders in Brazil.

## Resumen

**Objetivo:**

Analizar la asociación entre la prevalencia del diagnóstico de depresión en adultos y la cobertura de servicios de salud en las Unidades Federativas de Brasil.

**Métodos:**

Estudio transversal con datos de la Encuesta Nacional de Salud de 2019. Se calcularon las razones de momios ajustadas (*odds ratio*, OR) y los intervalos de confianza del 95 % (IC95%) mediante modelos de regresión logística multinivel para analizar la asociación entre el diagnóstico de depresión y la cobertura de servicios de salud.

**Resultados:**

En 2019 se analizaron 88.531 adultos residentes en las 27 Unidades Federativas brasileñas. Se observó una mayor probabilidad de diagnóstico de depresión entre los adultos residentes en Unidades Federativas con mayor cobertura de la Estrategia de Salud Familiar (OR 1,35; IC95% 1,29; 1,42), y con un mayor número de centros de apoyo a la salud familiar (OR 1,17; IC95% 1,13; 1,21) y centros de atención psicosocial (OR 1,31; IC95% 1,23; 1,41).

**Conclusión:**

Los resultados de este estudio indicaron que mayor cobertura de los servicios de salud se asoció con mayor prevalencia de diagnósticos de depresión. Estos hallazgos refuerzan la importancia de ampliar y fortalecer la red de atención psicosocial para mejorar el diagnóstico y la atención de las personas con trastornos mentales en Brasil.

**Ethical aspects**

This research used public domain anonymized databases.

## Introduction

Depression is a debilitating mental disorder that affects countless individuals in their daily activities, such as work, studies, social and family relationships, and is considered a global public health problem (1). In Brazil, in 2019, self-reported prevalence in adults approached 10%, with higher occurrence in women (15%) compared to men (5%) (2).

Depression is a mood disorder with a multifactorial etiology, resulting from the interaction of genetic and environmental factors (3-4). Elements that increase vulnerability include family history, stress, previous disorders, substance abuse, chronic diseases and grief (5). Clinically, depression can present isolated or combined symptoms, including sadness, low self-esteem and impaired concentration, which compromises daily routines and functional capacity (6). Although it is predominantly a psychological condition, evidence points to neurochemical alterations in the central nervous system, involving serotonin, norepinephrine and dopamine (7-8).

Depression, often called the “illness of the century”, has a broad impact on health and is associated with immunosuppression, inflammation, poor eating and hygiene habits, and can worsen other clinical conditions (5). Behaviors such as sedentary lifestyle, tobacco smoking and excessive alcohol consumption are more prevalent in people diagnosed with depression (2).

In Brazil, depressive disorders generate significant impacts not only on individual health, but also on the economic and public health spheres (9). Between 1990 and 2019, mental disorders were the main causes of years lived with disability (10), being one of the main causes of work absenteeism in the country. In addition, stigma associated with mental illnesses often hinders the search for treatment, which contributes to the worsening of the effects of depression.

Identifying and analyzing factors associated with depression are fundamental for informing public health policies and interventions that promote early diagnosis and expand access to treatment, especially among more vulnerable groups. In this sense, it becomes relevant to investigate the individual aspects and contextual characteristics that can influence diagnosis and management of this condition.

Despite the existence of comprehensive national data, gaps persist in understanding the role of health service coverage and other factors in the occurrence and identification of depression in Brazil. Multilevel analysis, by enabling the separation and quantification of individual and contextual effects, presents itself as an appropriate methodological tool to explore these relationships, contributing to the advancement of knowledge about the determinants of depression and to the formulation of more effective prevention and care strategies in the country.

The objective of this study was to analyze association between the prevalence of depression diagnosis in adults and health service coverage in Brazil’s Federative Units, using data from the 2019 National Health Survey (*Pesquisa Nacional de Saúde*, PNS).

## Methods

### Design and data source

This is a population-based cross-sectional study that used data from the 2019 PNS. The analysis adopted a multilevel approach to investigate association between diagnosis of depression and health service coverage in adults (≥18 years) residing in Brazil’s 27 Federative Units.

### Study setting, participants and size

The PNS was derived from a probabilistic sample representative of the Brazilian population (≥15 years) stratified into three stages: census tracts, households and residents (11).

Of the 108,525 households selected, 90,846 individuals were interviewed, resulting in a 16.2% non-response rate. For the purposes of this study, adults residing in Brazilian Federative Units were included; this resulted in a sample of 88,531 individuals. Data were obtained through household interviews, which collected information on sociodemographic and economic characteristics, lifestyle and presence of diseases (11).

The PNS was coordinated by the Brazilian Institute of Geography and Statistics and received approval from the National Research Ethics Committee (Opinion No. 3,529,376/2019) and the National Health Council. Informed consent was obtained from all participants included in the study (11).

### Variables

The dependent variable was the presence of depression, determined by individuals self-reporting having received a medical diagnosis, based on the following question: “Has any doctor or mental health professional (such as a psychiatrist or psychologist) ever diagnosed you with depression?” (yes, no).

At the individual level, the variables included were related to demographic and socioeconomic characteristics, lifestyle, presence of diseases, household registration with the Family Health Strategy (*Estratégia Saúde da Família*, ESF), and presence of private health insurance. Information was included on sex (male, female), age group (18-24, 25-39, 40-59, 60+ years), race/skin color (White, Brown [Brazilian mixed race], Black), schooling (incomplete elementary education, complete elementary education, complete high school education, complete higher education) – schooling being used as representative of individual socioeconomic status – and marital status (single, married, divorced widowed).

Lifestyle and presence of diseases were assessed based on information about alcohol consumption frequency (never, less than once a month, once or more times a month), regular physical activity (≥150 minutes/week), tobacco smoking (never smoked, former smoker, current smoker), and self-reported medical diagnosis of hypertension, diabetes, chronic kidney disease and chronic obstructive pulmonary disease.

The variables used to assess health service coverage were: household registration with the ESF, frequency of ESF household visits in the last year (never received a visit, monthly, bimonthly, less frequent), and presence of private health insurance (yes, no).

At the contextual level, factors related to health service coverage in the area of ​​residence were analyzed, obtained from the National Registry of Health Establishments and calculated for each federative unit in 2019. The contextual factors of interest included: ESF coverage (classified based on the percentage of the population covered by the service), number of active family health support centers (all modalities), number of active psychosocial care centers (all modalities), and per capita income.

Due to the non-linearity of the contextual variables, these were divided into quartiles, with the first quartile representing the lowest levels and the fourth quartile the highest levels of coverage.

### Statistical analysis and bias

Prevalence of depression diagnosis was estimated as the proportion of individuals who reported medical diagnosis in relation to the total sample, considering the weighting of the complex design of the PNS. In order to assess association between presence of depression and individual-level variables, we used Pearson’s chi-square test adjusted by Rao-Scott correction.

In the multivariate analysis, adjusted odds ratios (OR) for diagnosis of depression were estimated, along with their 95% confidence intervals (95%CI), using multilevel logistic regression models. This approach allowed for assessment of differences in outcome probability associated with individual variables, controlling for the effect of the aggregation level of the Federative Units. Odds ratios were estimated following a hierarchical approach, in which variables were progressively incorporated into the models. The conceptual framework adopted (12) began with sociodemographic characteristics, then lifestyle, then presence of diseases, and incorporated health service coverage.

In order to analyze the effect of contextual variables related to health service coverage, we used models adjusted for individual factors and per capita income of the Federative Unit of residence. The inclusion of contextual per capita income allowed for controlling population variation and the socioeconomic characteristics of the federative units, considering the heterogeneity in size and demographic profile of the territories and reducing possible biases arising from this variation. The effects of coverage by the ESF, family health support centers and psychosocial care centers were assessed independently of each other and enabled identification of the isolated contribution of each coverage modality to the prevalence of depression.

The model parameters were estimated by Bayesian inference, an approach recommended to reduce biases associated with maximum likelihood estimation in multilevel analyses (13). The intraclass correlation coefficient was also estimated to quantify the proportion of the outcome variance that can be explained by the contextual and individual levels.

The descriptive analyses considered sample weights and grouping by Federative Unit. Multilevel models were estimated using gllamm and incorporated sample weights according to the complex sample design. All analyses were conducted using Stata v14.2.

## Results

The bivariate analysis identified significant associations between presence of depression and individual characteristics (Table 1). Higher prevalence of depression was observed in females (14.7%), in older age groups, in the White race/skin color group (12.5%) and in divorced/widowed individuals (32.1%). Physically active adults showed lower prevalence of depression (9.3%), while former smokers (11.8%) and current smokers showed higher prevalence (11.5%).

**Table 1.** Distribution of demographic, socioeconomic, lifestyle, disease presence and healthcare access characteristics of the Brazilian adult population, according to self-reported diagnosis of depression. Brazil, 2019 (n=88,531)

Variables	Prevalence of depression n (%)	
Total	8,242 (10.2)	
Sex		
Male	1,930 (5.1)	<0.001
Female	6,312 (14.7)	
Age group (years)		
18–24	402 (5.9)	<0.001
25–39	1,805 (8.1)	
40–59	3,669 (12.7)	
≥60	2,336 (11.8)	
Race/skin color		
White	3,796 (12.5)	<0.001
Brown	3,573 (8.6)	
Black	766 (8.2)	
Schooling		
Incomplete elementary education	3,269 (10.9)	<0.001
Complete elementary education	996 (9.4)	
Complete high school education	2,363 (9.0)	
Complete higher education	1,614 (12.2)	
Marital status		
Single	2,961 (8.4)	<0.001
Married	3,119 (10.1)	
Divorced	1,151 (17.9)	
Widowed	1,011 (14.2)	
Alcohol consumption frequency		
Never	5,566 (11.6)	<0.001
Less than once a month	910 (9.1)	
Once or more times a month	1,766 (8.0)	
Regular physical activity (≥150 minutes/week)		
No	6,163 (10.6)	0.004
Yes	2,079 (9.3)	
Tobacco smoking		
Never smoked	4,436 (9.3)	<0.001
Former smoker	2,545 (11.8)	
Current smoker	1,231 (11.5)	
Presence of hypertension		
No	4,853 (8.7)	<0.001
Yes	3,355 (15.3)	
Presence of diabetes		
No	7,033 (10.3)	<0.001
Yes	1,032 (15.4)	
Presence of chronic kidney disease		
No	7,993 (10.1)	<0.001
Yes	249 (21.1)	
Presence of chronic obstructive pulmonary disease		
No	7,946 (9.9)	<0.001
Yes	962 (27.3)	
Household registered with the Family Health Strategy (ESF)		
No	2,069 (10.1)	0.771
Yes	5,257 (10.3)	
Frequency of ESF household visits in the last year		
Never received a visit	1,383 (11.5)	0.015
Monthly	1,902 (9.6)	
Bimonthly or less	1,972 (10.3)	
Presence of private health insurance		
No	5,739 (9.3)	<0.001
Yes	2,503 (12.7)	

Higher depression prevalence rates were observed in people with hypertension (15.3%), diabetes (15.4%), chronic kidney disease (21.1%), and/or chronic obstructive pulmonary disease (27.3%). Individuals who received regular visits from the ESF teams had lower prevalence of depression (9.6%). Prevalence of depression was higher among those with private health insurance (12.7%) (Table 1).

The results of the multilevel logistic regression models for presence of depression in adults, considering individual characteristics, are shown in Table 2. Females (OR 3.16; 95%CI 2.86; 3.49) were more likely to have diagnosis of depression compared to males. People who self-identified as being of Brown or Black race/skin color had lower odds of having diagnosis of depression (OR 0.86; 95%CI 0.82; 0.92 and OR 0.77; 95%CI 0.68; 0.88) compared to White individuals. Divorced individuals were more likely to have depression compared to single individuals (OR 1.48; 95%CI 1.37; 1.61). Alcohol consumption was associated with lower odds of depression diagnosis (OR 0.73; 95%CI 0.64; 0.82), while tobacco smoking was associated with higher odds (OR 1.43; 95%CI 1.25; 1.64).

**Table 2:** Odds ratios (OR) and 95% confidence intervals (95%CI) of prevalence of depression among adults, according to demographic, socioeconomic, lifestyle, disease presence and health service coverage characteristics. Brazil, 2019 (n=88,531)

Variables	Model 1a OR (95%CI)	Model 2b OR (95%CI)
Sex		
Male	1.00	1.00
Female	3.16d (2.86; 3.49)	3.07d (2.65; 3.56)
Age group (years)		
18–24	1.00	1.00
25–39	1.33c (1.10; 1.60)	1.39 (0.85; 1.52)
40–59	2.06d (1.66; 2.57)	1.58c (1.17; 2.18)
≥60	1.83d (1.51; 2.22)	1.01 (0.71; 1.44)
Race/skin color		
White	1.00	1.00
Brown	0.86d (0.82; 0.91)	0.82d (0.77; 0.88)
Black	0.77d (0.68; 0.88)	0.71d (0.59; 0.86)
Schooling		
Incomplete elementary education	1.00	1.00
Complete elementary education	0.95 (0.79; 1.15)	0.96 (0.78; 1.19)
Complete high school education	0.89c (0.81; 0.98)	0.89 (0.77; 1.03)
Complete higher education	1.03 (0.89; 1.19)	0.91 (0.80; 1.04)
Marital status		
Single	1.00	1.00
Married	0.96 (0.89; 1.03)	0.93 (0.84; 1.03)
Divorced	1.48d (1.37; 1.61)	1.58d (1.40; 1.78)
Widowed	0.94 (0.83; 1.05)	0.80c (0.66; 0.97)
Alcohol consumption frequency		
Never	1.00	
Less than once a month	0.73d (0.64; 0.82)	
Once or more times a month	0.72d (0.60; 0.87)	
Regular physical activity (≥150 minutes/week)		
No	1.00	
Yes	0.96 (0.89; 1.04)	
Tobacco smoking		
Never smoked	1.00	
Former smoker	1.33d (1.18; 1.50)	
Current smoker	1.43d (1.25; 1.64)	
Presence of hypertension		
No	1.00	
Yes	1.49d (1.38; 1.62)	
Presence of diabetes		
No	1.00	
Yes	1.18d (1.08; 1.31)	
Presence of chronic kidney disease		
No	1.00	
Yes	1.50c (1.15; 1.95)	
Presence of chronic obstructive pulmonary disease		
No	1.00	
Yes	2.47d (2.08; 2.94)	
Frequency of Family Health Strategy household visits in the last year		
Never received a visit	1.00	
Monthly	0.83c (0.74; 0.93)	
Bimonthly or less	0.94c (0.88; 0.99)	
Presence of private health insurance		
No	1.00	
Yes	1.17c (1.04; 1.33)	
Bayesian information criterion	96.189.144	56.066.876
Intraclass correlation coefficient	0.0268	0.0196

^a^Model adjusted with the demographic and socioeconomic variables; ^b^Model adjusted with the demographic, socioeconomic, lifestyle, presence of diseases and health service coverage variables; ^c^p-valor<0,050; ^d^p-valor≤0,001.

Presence of chronic diseases was consistently associated with higher odds of depression in adults, including hypertension, diabetes, chronic kidney disease and chronic obstructive pulmonary disease. Results are also presented regarding association between depression and care provided by the ESF and by private health insurance (Table 2). Regular ESF home visits were associated with lower odds of depression (OR 0.83; 95%CI 0.74; 0.93). Having private health insurance was associated with higher odds of diagnosis of depression (OR 1.17; 95%CI 1.04; 1.33).

The results of association between presence of depression and health service coverage, adjusted for individual factors, are shown in Table 3.

**Table 3:** Odds ratios (OR) and 95% confidence intervals (95%CI) of prevalence of depression among adults, adjusted for individual characteristicsa and per capita income of federative unit of residence, according to Family Health Strategy coverage (model 1), Family Health Support Center coverage (model 2) and Psychosocial Care Center coverage (model 3). Brazil, 2019 (n=88,531)

Variables	Model 1 OR (95%CI)	Model 2 OR (95%CI)	Model 3 OR (95%CI)
Second level: Federative Unit of residence			
Family Healtd Strategy coverage, quartile			
1	1.00		
2	1.54^b^ (1.50; 1.58)		
3	1.78^b^ (1.71; 1.85)		
4	1.35^b^ (1.29; 1.42)		
Family Health Support Center coverage, quartile			
1		1.00	
2		0.97 (0.93; 1.01)	
3		2.01^b^ (1.91; 2.09)	
4		1.17^b^ (1.13; 1.21)	
Psychosocial Care Center coverage, quartile			
1			1.00
2			1.26^b^ (1.15; 1.38)
3			1.09^c^ (1.01; 1.19)
4			1.31^b^ (1.23; 1.41)
Per capita income, quartile			
1	1.00	1.00	1.00
2	1.27^b^ (1.20; 1.33)	1.05 (0.98; 1.11)	2.18^b^ (1.99; 2.38)
3	1.82^b^ (1.73; 1.91)	2.15^b^ (2.02; 2.29)	2.23^b^ (2.09; 2.38)
4	2.33^b^ (2.18; 2.49)	1.77^b^ (1.64; 1.91)	1.78^c^ (1.64; 1.92)
Bayesian information criterion	56.044.227	56.047.232	56.055.753
Intraclass correlation coefficient	0.0034	0.0081	0.0233

^a^ Individual characteristics: sex; age group; race/skin color; schooling; marital status; alcohol consumption frequency; regular physical activity; tobacco smoking; presence of hypertension; diabetes; chronic kidney disease; chronic obstructive pulmonary disease; household registered with the Family Health Strategy; presence of private health insurance; ^b^p-valor<0,050; ^c^p-valor≤0,001.

Adults residing in areas with greater ESF coverage had higher odds of depression in the second (OR 1.54; 95%CI 1.50; 1.58), third (OR 1.78; 95%CI 1.71; 1.85), and fourth quartiles (OR 1.35; 95%CI 1.29; 1.42). Greater health support center coverage was associated with higher prevalence of depression, even after controlling for individual characteristics and per capita income (OR 1.17; 95%CI 1.13; 1.21) (Table 3).

There was higher prevalence of depression in areas with greater psychosocial care center coverage. Compared to adults living in areas with lower coverage, those in the second (OR 1.26; 95%CI 1.15; 1.38), third (OR 1.09; 95%CI 1.01; 1.19) and fourth quartiles (OR 1.31; 95%CI 1.23; 1.41) had higher odds of depression (Table 3).

## Discussion

This study identified that expanded health service coverage, especially ESF, family health support centers and psychosocial care centers, was significantly associated with higher prevalence rates of diagnosis of depression in Brazilian adults. The findings showed that the odds of diagnosis of depression were higher in areas with greater coverage of these services, suggesting that the expansion and integration of the mental health care network favors identification and monitoring of depression cases in the population.

The ESF has played a fundamental role in strengthening the Brazilian Unified Health System (*Sistema Único de Saúde*, SUS), being associated with improvements in vital statistics and increased disease cure rates (14). Since the creation of the SUS, ESF coverage has been expanded and several activities have been almost universalized, which has provided greater access to mental health care, especially for populations of lower socioeconomic status.

The association between greater health service coverage and greater prevalence of depression diagnosis may reflect the fundamental role of facilitated access in the early and appropriate identification of the disorder. Expansion of the ESF, family health support centers and psychosocial care centers strengthens the primary care network and matrix support care, which facilitates case detection and follow-up (15).

This suggests that the increase in prevalence observed in areas with greater coverage may reflect, in part, greater capacity to detect cases and not necessarily an actual increase in the prevalence of the disease. In this sense, adequate structuring of the psychosocial care network is fundamental for expanding the population’s access to mental health services, which favors identification of disorders in the most diverse territories, in addition to comprehensive and continuous care (18)

The ESF is the main entry point to the SUS and plays a strategic role in the early identification of depressive disorders. Mental health care in primary care allows for continuous and collaborative interventions, based on the interaction between professionals and users, which favors more integrated, humanized and effective care (16). Family health support centers expand the multidisciplinary approach of the ESF teams, promoting support and contributing to diagnosis and management of more complex cases. Psychosocial care centers are specialized mental health services, focused on severe and persistent disorders, and act as a tool to optimize the functioning of the psychosocial care network (17).

The results of this study indicated that places with a higher number of psychosocial care centers presented higher prevalence of diagnosis of depression, suggesting that territories with specialized support can favor both identification of the disorder and its care. Although psychosocial care centers do not provide direct care for common mental disorders, they offer matrix support to other services in the network, which contributes positively to the management of these conditions.

In addition to the influence of contextual factors, individual characteristics were shown to be associated with presence of depression, in line with recent national studies (2,19). Higher prevalence of the condition was observed among females, middle-aged adults, White people, individuals with lower schooling, divorced people, smokers, people with chronic diseases, and those with private health insurance. These findings reinforced the vulnerability profile already described in the literature and highlighted the importance of care strategies that consider the social determinants and clinical specificities of the population served.

Higher prevalence of depression among females can be explained by the complex interaction of biopsychosocial factors that begin in adolescence. These factors include specific hormonal changes during pubertal transition and exposure to psychosocial and occupational stressors throughout life, which favor the development of mental disorders, in addition to greater likelihood of females seeking health care and diagnoses (20,29). Higher prevalence of the condition was also observed among individuals aged 40-59 years, an age group marked by multiple biopsychosocial demands, including professional responsibilities, physical strain and financial worries (21).

The lower prevalence of depression among individuals of Black and Brown race/skin color compared to White individuals stands out, which may reflect inequalities in access to mental health diagnoses and treatments, resulting in underreporting of these disorders in the Black population (22). This difference is also related to lower health service coverage in areas with a higher concentration of these populations, since, in general, the Black and low-income population tends to reside in peripheral areas, characterized by barriers to access to public services and lower availability of specialized care services (32). On the other hand, lower schooling was associated with higher prevalence of depression, which can be explained by less access to information, financial instability and increased exposure to stressors (23).

This context reinforces the need for public policies that expand and improve the psychosocial care network in territories with a higher concentration of Black and Brown populations and lower levels of education, reducing gaps in care and promoting equity in the diagnosis and care of mental disorders. Divorced individuals also showed a higher prevalence of the condition compared to single individuals, suggesting the influence of emotional distress, stress and reduced social support resulting from the end of a marital relationship on the increased odds of developing depression (24).

Behaviors such as alcohol use and tobacco smoking have also been associated with depression, indicating the need for integrated approaches to managing these conditions. The findings of this study indicated lower diagnosis of depression among alcohol consumers, but this association should be interpreted with caution, since such a relationship can be largely explained by confounding factors such as socioeconomic level, physical health, as well as other risk behaviors. After adjusting for these factors, association between alcohol consumption and depression generally disappears and suggests that there is no robust evidence of a direct causal link between alcohol consumption and depressive symptoms (25). Regarding tobacco smoking, the higher prevalence of depression among those who used to smoke or still smoke corroborates the evidence between nicotine exposure and depressive symptoms (26).

Presence of chronic diseases, such as hypertension, diabetes, chronic kidney disease and chronic obstructive pulmonary disease, has been associated with higher prevalence of depression. These conditions increase psychological vulnerability due to their physical, emotional and social impacts, in addition to hindering adherence to treatment and raising stress levels (27). Identifying these factors along with a wide-ranging and accessible care network enhances early diagnosis and continuous care, especially among more vulnerable groups.

Socioeconomic status is recognized as a determinant of health and influences access to treatment and the quality of care for chronic diseases in Brazil (30). Having private health insurance can facilitate detection and management of depression, especially in contexts where public services face limitations in access or quality. Despite advances in reducing health inequalities in recent decades, the findings of this study suggested that such inequities may still persist in Brazil, manifesting themselves through differentiated access to and use of health services and consequently implying higher probability of diagnosis of depression among those who have private insurance (31).

Some limitations should be noted in this study. Despite methodological advances and the robustness of the sample, the interpretation of the results considered limitations inherent to the cross-sectional design of the study, which did not allow for inferring causality about the associations found. On the one hand, it is possible that greater health service coverage has facilitated identification and early diagnosis of depression, thus increasing the number of recorded cases. On the other hand, there is the possibility of reverse causality, that is, that regions with higher prevalence of depression have received greater investment in service coverage to meet this demand, a phenomenon that this study could not distinguish.

The impossibility of assessing the quality of services was considered to be another limitation for applying the results of this study, since it could vary significantly between regions, influencing both diagnosis and management of depression. The use of self-reported diagnosis, in addition to being subject to information bias and underreporting, limited the distinction between degrees of depression severity, which could modulate the intensity of association with service coverage, varying according to the severity of the clinical picture and access to specialized services.

Despite a 96.5% response rate, response bias could not be ruled out. Although the use of variables at different hierarchical levels may have exposed the study to the risk of aggregation bias, using multilevel analysis is a widely used technique to mitigate this type of bias, and is a strength of this investigation. This approach allowed for the separation of effects at the individual level from those attributable to context, thereby reducing misinterpretations.

The findings of this study indicated that greater health services coverage was associated with higher prevalence of diagnosis of depression, suggesting that expanding the psychosocial care network can support early identification and improve care for individuals with mental disorders in Brazil. Expanding this network and strengthening services such as the ESF, family health support centers and psychosocial care centers play a fundamental role in ensuring access to depression diagnosis and treatment.

Expansion and strengthening of the psychosocial care network must be conducted strategically, considering regional specificities and the different levels of health service coverage in the country. In Federative Units with lower coverage, it is essential to prioritize expanding access to basic services, such as the ESF and family health support centers, and to reinforce multidisciplinary support and psychosocial care in underserved areas. In regions with higher coverage, improving the quality of care, for example, through the efficient integration of psychosocial care centers with other network components, in addition to promoting longitudinal care, can improve the quality of mental health care in the territory. In this way, health service managers can direct resources and actions according to the specific needs of each context, contributing to reducing inequalities in access to and quality of health services.

## Data Availability

The data used in this article are available at: https://osf.io/gezyd/?view_only=44c38373ccd544a2bfe946c53fa70c30.
