# Supplementary material for: Association between diagnosis of depression and health service coverage: a multilevel analysis of the National Health Survey, Brazil, 2019
Source: Epidemiol Serv Saude. 2026 Feb 27;35:e20250666. doi: 10.1590/S2237-96222026v35e20250666.en (PMC12948296; doi:10.1590/S2237-96222026v35e20250666.en)
Supplement: Translation

# Translation: Associação entre diagnóstico de depressão e cobertura por serviços de saúde: análise multinível da Pesquisa Nacional de Saúde, Brasil, 2019

**DOI:** 10.1590/S2237-96222026v35e20250666.pt

**Authors:** Flávia Oliveira da Silva, Andressa Lourenço Carvalho, Guilherme Guimarães Maia Schnepper, Léo Domingues Marchesi, Isabella Ribeiro Leite, Laura Vazarin Endo, Camila Del Valhe Sanchez Lima, Kaio Henrique Correa Massa

**Affiliations:** 1 Universidade Anhembi Morumbi, São Paulo, SP, Brasil

**Keywords:** Depressão, Acesso a Serviços de Saúde, Determinantes Sociais da Saúde, Saúde Mental, Epidemiologia

## Abstract

**Objetivo:**

Analisar a associação entre a prevalência de diagnóstico de depressão em adultos e a cobertura de serviços de saúde nas Unidades Federativas do Brasil.

**Métodos:**

Estudo transversal utilizando dados da Pesquisa Nacional de Saúde 2019. Razão de chances (*odds ratio*, OR) ajustadas e intervalo de confiança de 95% (IC95%) foram calculados a partir de modelos de regressão logísticos multinível para analisar a associação entre diagnóstico de depressão e cobertura de serviços de saúde.

**Resultados:**

Foram analisados 88.531 adultos residentes das 27 Unidades Federativas brasileiras em 2019. Foi observada maior chance de diagnóstico de depressão entre adultos residentes em Unidades Federativas com maior cobertura pela Estratégia Saúde da Família (OR 1,35; IC95% 1,29; 1,42), e com maior número de núcleos de apoio à saúde da família (OR 1,17; IC95% 1,13; 1,21) e de centros de atenção psicossocial (OR 1,31; IC95% 1,23; 1,41).

**Conclusão:**

Os resultados deste estudo indicaram que a maior cobertura por serviços de saúde esteve associada à maior prevalência de diagnóstico de depressão. Estes achados reforçaram a importância da expansão e do fortalecimento da rede de atenção psicossocial para melhorar o diagnóstico e o cuidado de indivíduos com transtornos mentais no Brasil.

**Aspectos éticos**

Esta pesquisa utilizou bancos de dados de domínio público e anonimizados.:

## Introdução

A depressão é um transtorno mental incapacitante que afeta inúmeros indivíduos em suas atividades cotidianas, como trabalho, estudos, relações sociais e familiares, e é considerado um problema de saúde pública mundial (1). No Brasil, em 2019, a prevalência autorrelatada em adultos aproximou-se de 10%, com maior ocorrência em mulheres (15%) quando comparada aos homens (5%) (2).

A depressão é um transtorno do humor com etiologia multifatorial, resultante da interação de fatores genéticos e ambientais (3-4). Elementos que aumentam a vulnerabilidade incluem histórico familiar, estresse, transtornos prévios, dependência química, doenças crônicas e luto (5). Clinicamente, a depressão pode apresentar sintomas isolados ou combinados, incluindo tristeza, baixa autoestima e déficit de concentração, o que compromete a rotina e a capacidade funcional (6). Embora seja predominantemente uma condição psicológica, evidências apontam para alterações neuroquímicas no sistema nervoso central, envolvendo serotonina, noradrenalina e dopamina (7-8).

A depressão, frequentemente denominada de “mal do século”, impacta amplamente a saúde e está associada a imunossupressão, inflamação, maus hábitos alimentares e de higiene, além de agravar outras condições clínicas (5). Comportamentos como sedentarismo, tabagismo e consumo excessivo de álcool são mais prevalentes em pessoas com diagnóstico de depressão (2). 

No Brasil, os transtornos depressivos geram impactos significativos não apenas na saúde individual, mas também nas esferas econômica e de saúde pública (9). Entre 1990 e 2019, os transtornos mentais foram as principais causas de anos vividos com incapacidade (10), sendo uma das principais causas de afastamento laboral no país. Além disso, o estigma associado às doenças mentais frequentemente dificulta a busca por tratamento, o que contribui para o agravamento dos efeitos da depressão.

A identificação e a análise dos fatores associados à depressão são fundamentais para subsidiar políticas e intervenções de saúde pública que favoreçam o diagnóstico precoce e ampliem o acesso ao tratamento, sobretudo entre grupos mais vulneráveis. Nesse sentido, torna-se relevante investigar os aspectos individuais e as características contextuais que podem influenciar o diagnóstico e o manejo dessa condição. 

Apesar da existência de dados nacionais abrangentes, ainda persistem lacunas no entendimento do papel da cobertura dos serviços de saúde e de outros fatores sobre a ocorrência e a identificação da depressão no Brasil. A análise multinível, ao possibilitar a separação e a quantificação dos efeitos individuais e contextuais, apresenta-se como ferramenta metodológica apropriada para explorar essas relações, o que contribui para o avanço do conhecimento sobre os determinantes da depressão e para a formulação de estratégias mais efetivas de prevenção e de cuidado no país.

O objetivo deste estudo foi analisar a associação entre a prevalência de diagnóstico de depressão em adultos e a cobertura de serviços de saúde nas Unidades Federativas do Brasil, por meio de dados da Pesquisa Nacional de Saúde (PNS) 2019.

## Métodos

### Delineamento do estudo e fonte de dados

Trata-se de estudo transversal de base populacional que utilizou dados da PNS 2019. A análise adotou abordagem multinível para investigar a associação entre diagnóstico de depressão e cobertura dos serviços de saúde em adultos (≥18 anos) residentes nas 27 Unidades Federativas do Brasil.

### Contexto, participantes e tamanho do estudo

A PNS foi constituída a partir da amostra probabilística representativa da população brasileira (≥15 anos) estratificada em três estágios: setores censitários, domicílios e moradores (11).

Dos 108.525 domicílios selecionados, 90.846 indivíduos foram entrevistados, o que resultou na taxa de não resposta de 16,2%. Para os fins deste estudo, foram incluídos adultos residentes nas Unidades Federativas brasileiras; isso resultou na amostra de 88.531 indivíduos. Os dados foram obtidos por meio de entrevistas domiciliares, que coletaram informações sobre características sociodemográficas e econômicas, estilo de vida e presença de doenças (11).

A PNS foi coordenada pelo Instituto Brasileiro de Geografia e Estatística e recebeu aprovação do Comitê Nacional de Ética em Pesquisa (Parecer n° 3.529.376/2019) e do Conselho Nacional de Saúde no Brasil. Para todos os participantes incluídos no estudo, foi obtido o consentimento informado (11).

### Variáveis

A variável dependente foi a presença de depressão, determinada pelo autorrelato do indivíduo sobre já ter recebido diagnóstico médico a partir da seguinte pergunta: “Algum médico ou profissional de saúde mental (como psiquiatra ou psicólogo) já lhe deu o diagnóstico de depressão?” (sim, não).

No nível individual, as variáveis incluídas estavam relacionadas às características demográficas e socioeconômicas, ao estilo de vida, à presença de doenças, ao domicílio cadastrado na Estratégia Saúde da Família (ESF) e à presença de plano de saúde privado. Foram incluídas informações sobre sexo (masculino, feminino), faixa etária (18-24, 25-39, 40-59, 60+ anos), raça/cor da pele (branca, parda, preta), escolaridade (ensino fundamental incompleto, fundamental completo, médio completo, superior completo) – esta utilizada como representante da condição socioeconômica individual – e estado civil (solteiro, casado, divorciado, viúvo).

O estilo de vida e a presença de doenças foram avaliados com base nas informações sobre frequência de consumo de álcool (nunca, menos de uma vez por mês, uma ou mais vezes por mês), prática regular de atividade física (≥150 minutos/semana), tabagismo (nunca fumou, ex-fumante, fumante atual) e diagnóstico médico autorreferido de hipertensão, diabetes, doença renal crônica e doença pulmonar obstrutiva crônica.

As variáveis utilizadas para cobertura dos serviços de saúde foram: cadastro domiciliar na ESF, frequência de visitas da ESF no último ano (nunca recebeu, mensal, bimestral, com menor frequência) e presença de plano de saúde privado (sim, não).

No nível contextual, foram analisados fatores relacionados à cobertura dos serviços de saúde na área de residência, obtidos do Cadastro Nacional de Estabelecimentos de Saúde e calculados para cada Unidade Federativa em 2019. Os fatores contextuais de interesse incluíram: cobertura da ESF (classificada com base na porcentagem da população coberta pelo serviço), número de núcleos de apoio à saúde da família ativos (todas as modalidades), número de centros de atenção psicossocial ativos (todas as modalidades) e renda per capita.

Devido à não linearidade das variáveis contextuais, essas foram divididas em quartis, sendo o primeiro quartil representante dos menores níveis e o quarto quartil dos maiores níveis de cobertura.

### Análise estatística e viés

A prevalência de diagnóstico de depressão foi estimada como a proporção de indivíduos que relataram diagnóstico médico em relação ao total da amostra, considerando a ponderação do desenho complexo da PNS. Para avaliar a associação entre a presença de depressão e as variáveis do nível individual, utilizou-se o teste do qui-quadrado de Pearson ajustado pela correção de Rao-Scott.

Para a análise multivariada, foram estimadas razões de chances (*odds ratio*, OR) ajustadas para o diagnóstico de depressão, acompanhadas de seus intervalos de confiança de 95% (IC95%), através de modelos de regressão logística multinível. Essa abordagem permitiu avaliar as diferenças na probabilidade do desfecho associadas às variáveis individuais, controlando o efeito do nível de agregação das Unidades Federativas. As razões de chances foram estimadas seguindo uma abordagem hierarquizada, na qual as variáveis são incorporadas progressivamente aos modelos. A estrutura conceitual adotada (12) iniciou-se pelas características sociodemográficas, passando pelo estilo de vida e pela presença de doenças, e incorporou a cobertura por serviços de saúde. 

Para analisar o efeito das variáveis contextuais relacionadas à cobertura por serviços de saúde, foram utilizados modelos ajustados para fatores individuais e para a renda per capita da unidade federativa de residência. A inclusão da renda per capita contextual permitiu controlar a variação populacional e as características socioeconômicas das Unidades Federativas, considerando a heterogeneidade em tamanho e perfil demográfico dos territórios e reduzindo possíveis vieses decorrentes dessa variação. Os efeitos da cobertura por ESF, núcleos de apoio à saúde da família e centros de atenção psicossocial foram avaliados de forma independente entre si e permitiram identificar a contribuição isolada de cada modalidade de cobertura na prevalência de depressão.

Os parâmetros dos modelos foram estimados por inferência bayesiana, abordagem recomendada para reduzir os vieses associados à estimação por máxima verossimilhança em análises multinível (13). O coeficiente de correlação intraclasse também foi estimado para quantificar a proporção da variância do desfecho que pode ser explicada pelos níveis contextual e individual.

As análises descritivas consideraram pesos amostrais e agrupamento por unidade federativa. Os modelos multiníveis foram estimados por meio do modo gllamm e incorporaram os pesos amostrais segundo o desenho complexo da amostra. Todas as análises foram conduzidas no Stata v14.2.

## Resultados

A análise bivariada identificou associações significativas entre presença de depressão e características individuais (Tabela 1). Observou-se maior prevalência de depressão nas mulheres (14,7%), nas faixas etárias elevadas, na raça/cor da pele branca (12,5%) e nos divorciados/viúvos (32,1%). Adultos fisicamente ativos apresentaram menor prevalência de depressão (9,3%), enquanto ex-fumantes (11,8%) e fumantes atuais mostraram maior prevalência (11,5%).

**Tabela 1:** Distribuição das características demográficas, socioeconômicas, estilo de vida, presença de doenças e acesso à saúde da população adulta brasileira, segundo a presença de diagnóstico autorreferido de depressão. Brasil, 2019 (n=88.531)

Variáveis	Prevalência de depressão	
	n (%)	p-valor
Total	8.242 (10,2)	
Sexo		<0,001
Masculino	1.930 (5,1)	
Feminino	6.312 (14,7)	
**Faixa etária** (anos)		<0,001
18-24	402 (5,9)	
25-39	1.805 (8,1)	
40-59	3.669 (12,7)	
≥60	2.336 (11,8)	
**Raça/cor da pele**		<0,001
Branca	3.796 (12,5)	
Parda	3.573 (8,6)	
Preta	766 (8,2)	
Escolaridade		<0,001
Ensino fundamental incompleto	3.269 (10,9)	
Ensino fundamental completo	996 (9,4)	
Ensino médio completo	2.363 (9,0)	
Ensino superior completo	1.614 (12,2)	
**Estado civil**		<0,001
Solteiro	2.961 (8,4)	
Casado	3.119 (10,1)	
Divorciado	1.151 (17,9)	
Viúvo	1.011 (14,2)	
**Frequência de consumo de álcool**		<0,001
Nunca	5.566 (11,6)	
Menos que uma vez por mês	910 (9,1)	
Uma ou mais vezes por mês	1.766 (8,0)	
**Prática Regular de Atividade Física** (≥ **150 minutos**/semana)		0,004
Não	6.163 (10,6)	
Sim	2.079 (9,3)	
Tabagismo		<0,001
Nunca fumou	4.436 (9,3)	
Ex-fumante	2.545 (11,8)	
Fumante atual	1.231 (11,5)	
**Presença de** hipertensão		<0,001
Não	4.853 (8,7)	
Sim	3.355 (15,3)	
**Presença de diabetes**		<0,001
Não	7.033 (10,3)	
Sim	1.032 (15,4)	
**Presença de doença renal crônica**		<0,001
Não	7.993 (10,1)	
Sim	249 (21,1)	
**Presença de Doença Pulmonar Obstrutiva Crônic**a		<0,001
Não	7.946 (9,9)	
Sim	962 (27,3)	
**Domicílio cadastrado na Estratégia Saúde da Família** (ESF)		0,771
Não	2.069 (10,1)	
Sim	5.257 (10,3)	
**Frequência de visitas da ESF no último ano**		0,015
Nunca recebeu	1.383 (11,5)	
Mensal	1.902 (9,6)	
Bimestral ou com menor frequência	1.972 (10,3)	
**Presença de plano de saúde privado**		<0,001
Não	5.739 (9,3)	
Sim	2.503 (12,7)	

Foram observadas maiores prevalências de depressão em pessoas com hipertensão (15,3%), diabetes (15,4%), doença renal crônica (21,1%) e/ou doença pulmonar obstrutiva crônica (27,3%). Indivíduos que recebiam visitas regulares das equipes da ESF apresentaram menor prevalência de depressão (9,6%). A prevalência de depressão foi maior entre os que tinham plano de saúde privado (12,7%) (Tabela 1).

Apresentaram-se os resultados dos modelos de regressão logística multinível para a presença de depressão em adultos, considerando características individuais (Tabela 2). As mulheres (OR 3,16; IC95% 2,86; 3,49) apresentaram maior probabilidade de diagnóstico de depressão em comparação aos homens. Pessoas que se autodeclararam pardas ou pretas apresentaram menores chances de diagnóstico de depressão (OR 0,86; IC95% 0,82; 0,92 e OR 0,77; IC95% 0,68; 0,88) em comparação às brancas. Os divorciados apresentaram maior probabilidade de depressão em comparação aos solteiros (OR 1,48; IC95% 1,37; 1,61). O consumo de álcool esteve associado a menores chances de diagnóstico de depressão (OR 0,73; IC95% 0,64; 0,82), e o tabagismo esteve relacionado a maiores probabilidades da doença (OR 1,43; IC95% 1,25; 1,64). 

**Tabela 2:** Razão de chances (*odds ratio*, OR) e intervalo de confiança de 95% (IC95%) da prevalência de depressão em adultos, segundo características demográficas, socioeconômicas, estilo de vida, presença de doenças e cobertura por serviços de saúde. Brasil, 2019 (n=88.531)

Variáveis	Modelo 1^a^	Modelo 2^b^
	OR (IC95%)	OR (IC95%)
Sexo		
Masculino	1,00	1,00
Feminino	3,16^d^ (2,86; 3,49)	3,07^d^ (2,65; 3,56)
**Faixa etária** (anos)		
18-24	1,00	1,00
25-39	1,33^c^ (1,10; 1,60)	1,39 (0,85; 1,52)
40-59	2,06^d^ (1,66; 2,57)	1,58^c^ (1,17; 2,18)
≥60	1,83^d^ (1,51; 2,22)	1,01 (0,71; 1,44)
**Raça/cor da pele**		
Branca	1,00	1,00
Parda	0,86^d^ (0,82; 0,91)	0,82^d^ (0,77; 0,88)
Preta	0,77^d^ (0,68; 0,88)	0,71^d^ (0,59; 0,86)
Escolaridade		
Ensino fundamental incompleto	1,00	1,00
Ensino fundamental completo	0,95 (0,79; 1,15)	0,96 (0,78; 1,19)
Ensino médio completo	0,89^c^ (0,81; 0,98)	0,89 (0,77; 1,03)
Ensino superior completo	1,03 (0,89; 1,19)	0,91 (0,80; 1,04)
**Estado civil**		
Solteiro	1,00	1,00
Casado	0,96 (0,89; 1,03)	0,93 (0,84; 1,03)
Divorciado	1,48^d^ (1,37; 1,61)	1,58^d^ (1,40; 1,78)
Viúvo	0,94 (0,83; 1,05)	0,80^c^ (0,66; 0,97)
**Frequência de consumo de álcool**		
Nunca		1,00
Menos que uma vez por mês		0,73^d^ (0,64; 0,82)
Uma ou mais vezes por mês		0,72^d^ (0,60; 0,87)
**Prática regular de atividade física** (≥**150 minutos**/semana)		
Não		1,00
Sim		0,96 (0,89; 1,04)
Tabagismo		
Nunca fumou		1,00
Ex-fumante		1,33^d^ (1,18; 1,50)
Fumante atual		1,43^d^ (1,25; 1,64)
**Presença de hipertensão**		
Não		1,00
Sim		1,49^d^ (1,38; 1,62)
**Presença de diabetes**		
Não		1,00
Sim		1,18^d^ (1,08; 1,31)
**Presença de doença renal crônica**		
Não		1,00
Sim		1,50^c^ (1,15; 1,95)
**Presença de doença pulmonar obstrutiva crônica**		
Não		1,00
Sim		2,47^d^ (2,08; 2,94)
**Frequência de visitas da Estratégia Saúde da Família no último ano**		
Nunca recebeu		1,00
Mensal		0,83^c^ (0,74; 0,93)
Bimestral ou com menor frequência		0,94^c^ (0,88; 0,99)
**Presença de plano de saúde privado**		
Não		1,00
Sim		1,17^c^ (1,04; 1,33)
Critério de informação bayesiano	96.189.144	56.066.876
**Coeficiente de correlação** intraclasse	0,0268	0,0196

^a^Modelo ajustado pelas variáveis demográficas e socioeconômicas; ^b^Modelo ajustado pelas variáveis demográficas, socioeconômicas, estilo de vida, presença de doenças e cobertura por serviços de saúde; ^c^p-valor<0,050; ^d^p-valor≤0,001.

A presença de doenças crônicas esteve consistentemente associada a maiores chances de depressão em adultos, incluindo hipertensão, diabetes, doença renal crônica e doença pulmonar obstrutiva crônica. Também foram apresentados resultados sobre a associação entre depressão e atendimento pela ESF e por plano de saúde (Tabela 2). Visitas domiciliares regulares da ESF estiveram associadas a menores chances de depressão (OR 0,83; IC95% 0,74; 0,93). Ter plano de saúde privado esteve associado a maiores probabilidades de diagnóstico de depressão (OR 1,17; IC95% 1,04; 1,33).

Foram mostrados os resultados da associação entre presença de depressão e cobertura dos serviços de saúde, ajustados para os fatores individuais (Tabela 3). 

**Tabela 3:** Razão de chances (*odds ratio*, OR) e intervalo de confiança de 95% (IC95%) da prevalência de depressão em adultos ajustadas por características individuais^a^ e renda per capita da unidade federativa de residência, segundo cobertura por Estratégia Saúde da Família (modelo 1), Núcleo de Apoio à Saúde da Família (modelo 2) e Centro de Atenção Psicossocial (modelo 3). Brasil, 2019 (n=88.531)

Variáveis	Modelo 1	Modelo 2	Modelo 3
	OR (IC95%)	OR (IC95%)	OR (IC95%)
**Segundo nível: Unidade Federativa de residência**			
**Cobertura por Estratégia Saúde da Família**, quartil			
1	1,00		
2	1,54^b^ (1,50; 1,58)		
3	1,78^b^ (1,71; 1,85)		
4	1,35^b^ (1,29; 1,42)		
**Cobertura por Núcleo de Apoio à Saúde da Família**, quartil			
1		1,00	
2		0,97 (0,93; 1,01)	
3		2,01^b^ (1,91; 2,09)	
4		1,17^b^ (1,13; 1,21)	
**Cobertura por Centro de Atenção Psicossocial**, quartil			
1			1,00
2			1,26^b^ (1,15; 1,38)
3			1,09^c^ (1,01; 1,19)
4			1,31^b^ (1,23; 1,41)
**Renda per capita**, quartil			
1	1,00	1,00	1,00
2	1,27^b^ (1,20; 1,33)	1,05 (0,98; 1,11)	2,18^b^ (1,99; 2,38)
3	1,82^b^ (1,73; 1,91)	2,15^b^ (2,02; 2,29)	2,23^b^ (2,09; 2,38)
4	2,33^b^ (2,18; 2,49)	1,77^b^ (1,64; 1,91)	1,78^c^ (1,64; 1,92)
**Critério de informação bayesiano**	56.044.227	56.047.232	56.055.753
**Coeficiente de correlação intraclasse**	0,0034	0,0081	0,0233

^a^Características individuais: sexo; faixa etária; raça/cor da pele; escolaridade; estado civil; frequência de consume de álcool; prática regular de atividade física; tabagismo; presença de hipertensão; diabetes; doença renal crônica; doença pulmonar obstrutiva crônica; domicílio cadastrado na Estratégia Saúde da Família; presença de plano de saúde privado; ^b^p-valor<0,050; ^c^p-valor≤0,001.

Adultos residentes em áreas com maior cobertura da ESF tiveram maiores chances de depressão nos segundo (OR 1,54; IC95% 1,50; 1,58), terceiro (OR 1,78; IC95% 1,71; 1,85) e quarto quartis (OR 1,35; IC95% 1,29; 1,42). A maior cobertura de núcleos de apoio à saúde associou-se à maior prevalência de depressão, mesmo após controle por características individuais e renda per capita (OR 1,17; IC95% 1,13; 1,21) (Tabela 3).

Houve maior prevalência de depressão nas áreas com a maior cobertura dos centros de atenção psicossocial. Comparado aos adultos que viviam nas áreas com menor cobertura, aqueles nos segundo (OR 1,26; IC95% 1,15; 1,38), terceiro (OR 1,09; IC95% 1,01; 1,19) e quarto quartis (OR 1,31; IC95% 1,23; 1,41) apresentaram maiores chances de depressão (Tabela 3).

## Discussão

Este estudo identificou que a cobertura ampliada por serviços de saúde, especialmente ESF, núcleos de apoio à saúde da família e centros de atenção psicossocial, esteve significativamente associada a maiores prevalências de diagnóstico de depressão em adultos brasileiros. Os achados evidenciaram que as chances de diagnóstico de depressão foram maiores em áreas com maior cobertura desses serviços, o que sugere que a ampliação e a integração da rede de atenção à saúde mental favorecem a identificação e o acompanhamento de casos de depressão na população.

A ESF tem desempenhado papel fundamental no fortalecimento do Sistema Único de Saúde, estando associada às melhorias nas estatísticas vitais e ao aumento das taxas de cura de doenças (14). Desde a criação do Sistema Único de Saúde, a cobertura da ESF foi ampliada e diversas atividades foram quase universalizadas, o que proporcionou maior acesso aos cuidados de saúde mental, especialmente para populações de nível socioeconômico mais baixo. 

A associação entre maior cobertura por serviços de saúde e maior prevalência do diagnóstico de depressão pode refletir o papel fundamental do acesso facilitado na identificação precoce e adequada do transtorno. A ampliação da ESF, de núcleos de apoio à saúde da família e de centros de atenção psicossocial fortalece a rede de atenção básica e o cuidado matricial, o que facilita a detecção e o acompanhamento dos casos (15). 

Isso sugere que o aumento da prevalência observada em áreas com maior cobertura pode refletir, em parte, a maior capacidade de detecção dos casos e não necessariamente o aumento real da prevalência da doença. Nesse sentido, a estruturação adequada da rede de atenção psicossocial é fundamental para ampliar o acesso da população aos serviços de saúde mental, o que favorece a identificação de transtornos nos mais diversos territórios, além do cuidado integral e contínuo (18).

A ESF é a principal porta de entrada do SUS e desempenha papel estratégico na identificação precoce de transtornos depressivos. O cuidado em saúde mental na atenção básica permite intervenções contínuas e colaborativas, baseadas na interação entre profissionais e usuários, o que favorece o cuidado mais integrado, humanizado e eficaz (16). Os núcleos de apoio à saúde da família ampliam a abordagem multidisciplinar das equipes da ESF, promovendo suporte e contribuindo para o diagnóstico e o manejo dos casos mais complexos. Os centros de atenção psicossocial são serviços especializados em saúde mental, focados em transtornos graves e persistentes, e atuam como ferramenta para otimizar o funcionamento da rede de atenção psicossocial (17). 

Os resultados deste estudo indicaram que localidades com maior número de centros de atenção psicossocial apresentaram maior prevalência de diagnóstico de depressão, o que sugere que territórios com suporte especializado podem favorecer tanto a identificação do transtorno quanto o seu cuidado. Embora os centros de atenção psicossocial não realizem o cuidado direto com transtornos mentais comuns, eles oferecem apoio matricial aos demais serviços da rede, o que contribui positivamente para o manejo dessas condições. 

Além da influência dos fatores contextuais, características individuais se mostraram associadas à presença de depressão, em consonância com estudos nacionais recentes (2,19). Observou-se maior prevalência da doença entre mulheres, adultos de meia-idade, pessoas brancas, indivíduos com menor escolaridade, divorciados, fumantes, portadores de doenças crônicas e detentores de planos privados de saúde. Esses achados reforçaram o perfil de vulnerabilidade já descrito na literatura e destacaram a importância de estratégias de cuidado que considerem os determinantes sociais e as especificidades clínicas da população atendida.

A prevalência maior de depressão entre mulheres pode ser explicada pela complexa interação de fatores biopsicossociais que se iniciam desde a adolescência. Esses fatores incluem alterações hormonais específicas da transição puberal e exposição a estressores psicossociais e ocupacionais ao longo da vida, os quais favorecem o desenvolvimento de transtornos mentais, além de haver maior probabilidade de busca por atendimento de saúde e por diagnósticos entre as mulheres (20,29). Observou-se também maior prevalência da doença entre indivíduos de 40-59 anos, faixa etária marcada por múltiplas demandas biopsicossociais, incluindo responsabilidades profissionais, desgastes físicos e preocupações financeiras (21). 

Destaca-se a menor prevalência de depressão entre indivíduos pretos e pardos em comparação aos brancos, o que pode refletir as desigualdades no acesso a diagnósticos e tratamentos de saúde mental, resultando em subnotificação desses transtornos na população negra (22). Essa diferença também está relacionada à menor cobertura dos serviços de saúde em áreas com maior concentração dessas populações, pois, de modo geral, a população negra e de baixa renda tende a residir em áreas periféricas, caracterizadas por barreiras no acesso aos serviços públicos e por menor disponibilidade de serviços de atenção especializada (32). Por outro lado, a menor escolaridade esteve associada à maior prevalência de depressão, o que pode ser explicado pelo menor acesso a informações, pela instabilidade financeira e pela exposição aumentada a fatores estressantes (23). 

Esse contexto reforça a necessidade de políticas públicas que ampliem e qualifiquem a rede de atenção psicossocial em territórios com maior concentração de população preta, parda e de menor escolaridade, reduzindo vazios assistenciais e promovendo a equidade no diagnóstico e no cuidado dos transtornos mentais. Indivíduos divorciados também apresentaram maior prevalência da doença em relação aos solteiros, sendo possível inferir a influência de desgaste emocional, estresse e redução de suporte social decorrentes do término da relação afetiva conjugal no aumento de chances de desenvolvimento de depressão (24).

Comportamentos como uso de álcool e tabagismo também estiveram associados à depressão, indicando a necessidade de abordagens integradas para o manejo dessas condições. Os achados deste estudo indicaram menor diagnóstico de depressão entre consumidores de álcool, mas essa associação deve ser interpretada com cautela, uma vez que tal relação pode ser explicada em grande parte por fatores de confusão, como nível socioeconômico, saúde física e outros comportamentos de risco. Após ajustes para esses fatores, a associação entre consumo de álcool e depressão geralmente desaparece e sugere que não há evidência robusta de ligação causal direta entre consumo de álcool e sintomas depressivos (25). Em relação ao tabagismo, a maior presença de depressão entre aqueles que já fumaram ou ainda fumam corrobora as evidências entre a exposição à nicotina e a sintomas depressivos (26).

A presença de doenças crônicas, como hipertensão, diabetes, doença renal crônica e doença pulmonar obstrutiva crônica, esteve associada à maior prevalência de depressão. Essas condições aumentam a vulnerabilidade psicológica devido aos seus impactos físicos, emocionais e sociais, além de dificultarem a adesão ao tratamento e elevarem os níveis de estresse (27). Identificar esses fatores junto a uma rede ampliada e acessível potencializa o diagnóstico precoce e o cuidado contínuo, sobretudo nos grupos mais vulneráveis.

O status socioeconômico é reconhecidamente um determinante da saúde e influencia o acesso ao tratamento e a qualidade do cuidado de doenças crônicas no Brasil (30). Possuir plano privado de saúde pode facilitar a detecção e o manejo da depressão, especialmente em contextos nos quais os serviços públicos enfrentam limitações de acesso ou de qualidade. Apesar dos avanços na redução das desigualdades em saúde nas últimas décadas, os achados deste estudo sugeriram que tais iniquidades ainda podem persistir no país, manifestando-se através de acessos e usos diferenciados aos serviços de saúde e implicando, consequentemente, uma maior probabilidade de diagnóstico de depressão entre aqueles que possuem planos privados (31).

Algumas limitações devem ser apontadas neste estudo. Apesar dos avanços metodológicos e da robustez da amostra, a interpretação dos resultados considerou limitações inerentes ao desenho transversal do estudo, que não permitiu inferir causalidade sobre as associações observadas. Por um lado, é possível que a maior cobertura de serviços de saúde tenha facilitado a identificação e o diagnóstico precoce da depressão, o que ampliou o número de casos registrados. Por outro lado, existe a possibilidade de causalidade reversa, ou seja, que regiões com maior prevalência de depressão tenham recebido maior investimento em cobertura por serviços para responder a essa demanda, fenômeno que este estudo não pôde distinguir. 

A impossibilidade de avaliar a qualidade dos serviços foi considerada outra limitação para aplicação dos resultados deste estudo, uma vez que ela pôde variar de forma importante entre regiões, influenciando tanto o diagnóstico quanto o manejo da depressão. A utilização do diagnóstico autorreferido, além de poder estar sujeito a viés de informação e subnotificação, limitou a distinção entre graus de severidade da depressão, o que pôde modular a intensidade da associação com a cobertura dos serviços, variando conforme a gravidade do quadro clínico e o acesso a serviços especializados. 

Apesar da taxa de resposta de 96,5%, o viés de respostas não pôde ser descartado. Embora o uso de variáveis em diferentes níveis hierárquicos possa ter exposto o estudo ao risco de viés por agregação, a adoção da análise multinível é uma técnica amplamente utilizada para mitigar esse tipo de viés, sendo um ponto forte da investigação. Essa abordagem permitiu separar os efeitos que pertenciam ao nível individual daqueles atribuíveis ao contexto, reduzindo interpretações equivocadas. 

Os achados deste estudo indicaram que a maior cobertura por serviços de saúde esteve associada à maior prevalência de diagnóstico de depressão, o que sugere que a expansão da rede de atenção psicossocial pode apoiar a identificação precoce e melhorar o cuidado de indivíduos com transtornos mentais no Brasil. A expansão dessa rede e o fortalecimento de serviços como a ESF, os núcleos de apoio à saúde da família e os centros de atenção psicossocial desempenham papel fundamental para garantir o acesso ao diagnóstico e ao tratamento da depressão.

A expansão e o fortalecimento da rede de atenção psicossocial devem ser conduzidos de forma estratégica, considerando as especificidades regionais e os diferentes níveis de cobertura de serviços de saúde no país. Nas Unidades Federativas com menor cobertura, é imprescindível priorizar a ampliação do acesso aos serviços básicos, como a ESF e os núcleos de apoio à saúde da família, e reforçar o suporte multidisciplinar e o cuidado psicossocial em territórios menos assistidos. Nas regiões com maior cobertura, o aprimoramento da qualidade do atendimento, por exemplo, a partir da integração eficiente dos centros de atenção psicossocial com os demais componentes da rede, além de promover o cuidado longitudinal, é capaz de melhorar a qualidade da assistência à saúde mental do território. Dessa maneira, os gestores poderão direcionar recursos e ações conforme as necessidades específicas de cada contexto, contribuindo para diminuir as desigualdades no acesso e na qualidade dos serviços de saúde.

## Data Availability

Os dados utilizados neste artigo estão disponíveis em: https://osf.io/gezyd/?view_only=44c38373ccd544a2bfe946c53fa70c30.
